# Supplementary material for: Water chemistry reveals a significant decline in coral calcification rates in the southern Red Sea
Source: Nat Commun. 2018 Sep 6;9:3615. doi: 10.1038/s41467-018-06030-6 (PMC6127156; doi:10.1038/s41467-018-06030-6)
Supplement: Supplementary file 1 — Supplementary Information [file 41467_2018_6030_MOESM1_ESM.pdf]

Supplementary information: **Water chemistry reveals a significant decline in coral calcification rates in the southern Red Sea**

Supplementary Table 1: Surface water samples processed in the present study. Concentrations of dissolved inorganic carbon and total alkalinity are reported for in situ salinities; concentrations of calcium and strontium are normalized to salinity of 35. Error bars reported for calcium and strontium mark the average deviation of duplicate analyses of the same sample from the mean.

Date + Time (mm/dd/yy UTC)	Region	Latitude Deg N	Longitude Deg E	Salinity	SST Deg C	DIC µmol kg^{-1}	A_T µmol kg^{-1}	Ca mmol kg^{-1}	Sr µmol kg^{-1}
12/24/15 11:00	Bay of Bengal	5.917	84.883	34.432		1935	2269	10.254 ±0.009	87.64 ±0.04
12/27/15 12:00	Arabian Sea	11.317	64.817	36.341		2016	2386	10.241 ±0.001	87.76 ±0.10
12/30/15 01:15	Gulf of Aden	12.767	47.550	36.187	26.000	2053	2371	10.225 ±0.003	87.81 ±0.07
12/30/15 16:15	Bab-el-Mandeb	12.617	43.367	35.959	26.473	2041	2352	10.230 ±0.006	87.95 ±0.06
12/30/15 21:10	Red Sea	13.717	42.583	36.315	26.035	2047	2371	10.243 ±0.011	88.20 ±0.01
12/31/15 02:00	Red Sea	14.900	41.900	36.754	26.419	2051	2383	10.235 ±0.001	88.11 ±0.02
12/31/15 08:00	Red Sea	16.367	41.067	37.332	26.896	2062	2411	10.223 ±0.005	88.18 ±0.01
12/31/15 14:15	Red Sea	17.867	40.217	37.998	28.766	2074	2441	10.219 ±0.005	88.05 ±0.08
12/31/15 18:10	Red Sea	18.800	39.667	37.782	28.527	2071	2436	10.228 ±0.011	88.13 ±0.09
12/31/15 21:50	Red Sea	19.700	39.133	38.199	28.247	2085	2453	10.210 ±0.002	87.94 ±0.03
01/01/16 02:10	Red Sea	20.717	38.550	39.456	27.992	2088	2483	10.191 ±0.001	87.70 ±0.04
01/01/16 08:00	Red Sea	22.050	37.767	39.378	27.146	2101	2505	10.208 ±0.011	87.90 ±0.05
01/01/16 12:00	Red Sea	23.000	37.200	40.024	25.942	2120	2511	10.189 ±0.002	87.65 ±0.02
01/01/16 16:40	Red Sea	24.117	36.533	40.115	26.040	2134	2512	10.187 ±0.001	87.85 ±0.04
01/01/16 21:55	Red Sea	25.333	35.667	40.145	24.973	2126	2508	10.191 ±0.000	87.88 ±0.02
01/02/16 02:15	Red Sea	26.250	34.783	40.365	25.367	2145	2499	10.201 ±0.007	88.10 ±0.03
01/02/16 08:00	Red Sea	27.083	34.100	40.191	26.000	2121	2506	10.199 ±0.013	87.84 ±0.10
04/18/16 11:15	Arabian Sea	12.467	60.433	36.530			2389		
04/19/16 12:00	Arabian Sea	13.817	52.950	35.720			2366		
04/20/16 06:30	Gulf of Aden	12.750	47.417	36.218			2363		
04/20/16 20:05	Bab-el-Mandeb	12.650	43.350	36.144			2373		
04/21/16 01:30	Red Sea	13.967	42.433	36.420			2373		
04/21/16 05:40	Red Sea	15.033	41.817	36.880			2385		
03/23/18	Bay of Bengal	6.055	86.293	33.344			2206	10.259±0.019	87.47±0.07
03/25/18	Indian ocean	8.677	72.980	34.091			2262	10.231±0.009	87.50±0.12

03/26/18	Arabian Sea	11.277	64.657	36.064	2369	10.220±0.001	87.41±0.17
03/29/18	Gulf of Aden	12.765	47.567	36.203	2375	10.210±0.019	87.70±0.11
03/29/18	Red Sea	12.997	43.187	36.625	2398	10.219±0.026	87.97±0.13
03/29/18	Red Sea	14.000	42.430	36.860	2400	10.223±0.027	87.98±0.13
03/30/18	Red Sea	14.962	42.017	37.047	2405	10.253±0.024	88.07±0.18
03/30/18	Red Sea	16.038	41.337	37.576	2420	10.216±0.031	87.96±0.17
03/30/18	Red Sea	17.007	40.723	37.434	2417	10.226±0.028	87.96±0.22
03/30/18	Red Sea	18.013	40.673	38.332	2447	10.237±0.022	87.79±0.28
03/30/18	Red Sea	19.002	39.440	38.353	2451	10.231±0.037	87.89±0.20
03/30/18	Red Sea	19.985	38.798	38.366	2438	10.185±0.021	87.85±0.18
03/31/18	Red Sea	21.022	38.123	38.850	2458	10.215±0.024	87.60±0.18

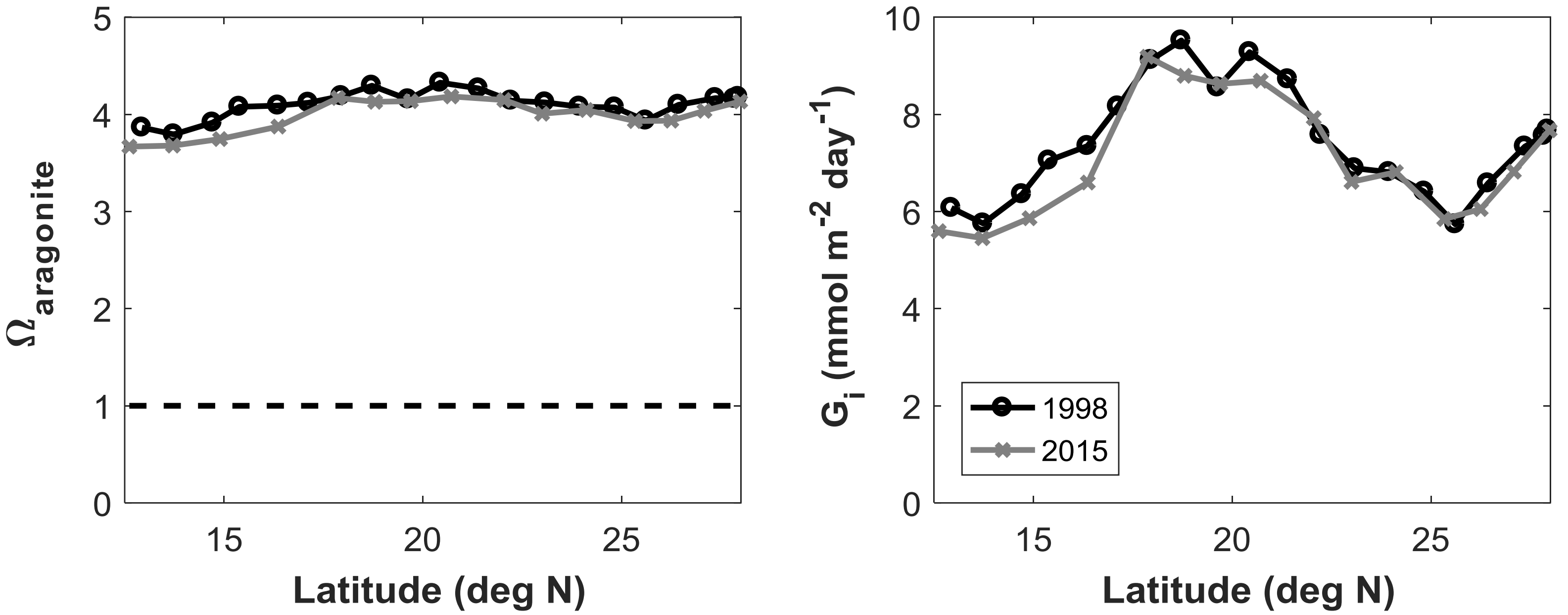

Supplementary Figure 1: Calculation of the effect of ocean acidification on coral calcification rates in the Red Sea during 1998-2015. Left panel depicts the calculated aragonite saturation state of Red Sea surface water, right panel shows calculated net coral reef community $CaCO_3$ deposition rates (G_i; right panel) in 1998 and 2015, assuming it had changed only due to acidification and warming. Dashed line at Ω=1 (left panel) marks saturation with respect to aragonite. The calculations assume equilibrium of Red Sea surface water with atmospheric CO_2 pressure as measured at Mauna Loa (ftp://aftp.cmdl.noaa.gov/products/trends/co2/co2_mm_mlo.txt) and warming of Red Sea surface water by 0.25°C during 1998-2015 (*1*). Calculations were done using the stoichiometric solubility product for aragonite in seawater from (*2*) and the Eilat reef equation (*3*, *4*). Carbonate ion concentrations were calculated using total alkalinity and pCO2 by CO2sys v2.1 (*5*) with K1, K2 from (*6*) and total boron concentrations from (*7*). Calcium concentrations were directly measured in the present study for the 2015 data and by (*8*) for the 1998 data.

Cited literature

1. V. Chaidez, D. Dreano, S. Agusti, C. M. Duarte, I. Hoteit, Decadal trends in Red Sea maximum surface temperature. *Scientific Reports* **7**, (2017).
2. A. Mucci, The solubility of calcite and aragonite in seawater at various salinities, temperatures, and one atmosphere total pressure. *American Journal of Science* **283**, 780-799 (1983).
3. J. Silverman, B. Lazar, J. Erez, Effect of aragonite saturation, temperature, and nutrients on the community calcification rate of a coral reef. *Journal of Geophysical Research-Oceans* **112**, (2007).
4. E. A. Burton, L. M. Walter, Relative precipitation rates of aragonite and Mg calcite from seawater: Temperature or carbonate ion control? *Geology* **15**, 111-114 (1987).
5. D. Pierrot, E. Lewis, D. W. R. Wallace. (ORNL/CDIAC-105a. Carbon Dioxide Information Analysis Center, Oak Ridge National Laboratory, U.S. Department of Energy, Oak Ridge, Tennessee, 2006).
6. C. Mehrbach, C. H. Culberson, J. E. Hawley, R. M. Pytkowicz, Measurement of the apparent dissociation constants of carbonic acid in seawater at atmospheric pressure. *Limnology and Oceanography* **18**, 897-907 (1973).
7. K. Lee *et al.*, The universal ratio of boron to chlorinity for the North Pacific and North Atlantic oceans. *Geochimica et Cosmochimica Acta* **74**, 1801-1811 (2010).
8. Z. Steiner *et al.*, Basin-scale estimates of pelagic and coral reef calcification in the Red Sea and Western Indian Ocean. *Proceedings of the National Academy of Sciences of the United States of America* **111**, 16303-16308 (2014).
